# Scientometrics Study of Research Output on Sheep and Goats from Greece

**DOI:** 10.3390/ani12192666

**Published:** 2022-10-04

**Authors:** Daphne T. Lianou, George C. Fthenakis

**Affiliations:** Veterinary Faculty, University of Thessaly, 43100 Karditsa, Greece

**Keywords:** goat, mastitis, meta-research, milk production, research analysis, research assessment, research mapping, sheep, small ruminants, veterinary

## Abstract

**Simple Summary:**

The article studies the research output on sheep and goats from Greece; a country where small ruminant farming is the most important terrestrial animal farming business. Milk production from sheep and goats exceeds that from cattle and over 90% of total milk produced from sheep and goats is used for dairy products. Research output on sheep and goats have increased significantly from 1997 to 2022. The bulk of publications, 87.5% of relevant papers, has originated from four establishments: Aristotle University of Thessaloniki; University of Thessaly; Agricultural University of Athens and Hellenic Agricultural Organization—Dimitra. Papers were published most frequently in the journals *Small Ruminant Research* and *Journal of the Hellenic Veterinary Medical Society*. The most frequent general topics of study were animal health-welfare and animal products. The findings have indicated that research has focused on milk production and diseases of the udder of small ruminants; moreover, there was accumulation of relevant research in Greece in some establishments only. The findings of the study can be used by researchers; stakeholders and Government entities to improve relevant research and to better allocate resources in the country.

**Abstract:**

The study is a scientometrics evaluation of published articles performed in Greece on sheep and goats during the last 25 years, a period coinciding with implementation of reforms to shape and consolidate tertiary education and research establishments in the country. Objectives were: evaluation of the relevant publications and presentation of quantitative characteristics regarding scientific content and bibliometric details. The Web of Science platform was used (search terms: [[*sheep* OR goat*] OR [*Ovis aries* OR *Capra hircus*]] (1997–2022)) and 1080 papers were considered in detail. Throughout the study period, there was a clear progressive increase in numbers of papers published. The papers originated from 39 different entities, most from Aristotle University of Thessaloniki (34.0%), University of Thessaly (28.0%), Agricultural University of Athens (21.2%) and Hellenic Agricultural Organization—Dimitra (13.6%). Papers were published in 318 different journals. Journals with more published papers were *Small Ruminant Research* and *Journal of the Hellenic Veterinary Medical Society*. The most frequent general topic of study in the papers was health and welfare (46.7% of papers); second most frequent topic was animal products (18.6%). The papers have received 16,558 citations, i.e., οn average 15.4 citations per paper; the *h*-index was 56, the *i_10_*-index was 518 and the yearly citations per paper were 1.71. Papers on goats had higher impact than papers on sheep. There were 1711 individual authors, of which 728 were first or last authors. In total, 24 authors have each co-authored ≥2.5% of all papers; five authors were each first or last in that proportion of all papers. The findings have indicated that relevant research has focused on milk production and diseases of the udder of small ruminants; moreover, there was accumulation of relevant research in Greece in some establishments only. The findings of the study can be employed to initiate improved relevant research approaches in the country.

## 1. Introduction

Scientometrics refers to ‘all quantitative aspects of science and scientific research’ [1] and can produce new knowledge through information available in previously published articles. Scientometrics methodologies can be useful in tracking changes in scientific research and technology and in evaluating respective output, by making a quantitative assessment of the relevant published articles. Moreover, the approach can be used to set research priorities, allocate funding and reward scientific excellence. Scientometrics papers differ from review papers, as the latter summarise and discuss knowledge on a topic, without reporting new facts or carrying out new analyses [2,3].

Internationally, there have been very limited scientometrics assessments of scientific work performed in the board fields of veterinary or animal science. An assessment of records in the Web of Science database after using the terms: [‘*scientometrics*’ AND (‘*veterinary*’ OR ‘*animal*’)] revealed only 12 published articles. Among these, Gupta et al. [4] evaluated the international research output on camels (3089 papers from 2003 to 2012), Freire and Nicol [5] presented the changes in the reporting on animal welfare during the range 1968 to 2017, whilst Gonzalez and Salgado-Arroyo [6] and Garg et al. [7] assessed the veterinary research output in Colombia (3000 papers from 2010 to 2019) and India (7056 papers from 2001 to 2020), respectively.

Greece has high numbers of sheep (approximately 8,400,000) and goats (approximately 3,600,000) [8], which account for around 6.5% and 22.0%, respectively, of the total number of small ruminants in Europe [9]. Small ruminant farming is the most important terrestrial animal farming business in Greece, generating 18% of the total primary sector income [10]. Milk production from sheep and goats amounts to 645,000 and 350,000 tons annually [9,11] and, in the country, those quantities exceed milk production of cattle [12]. Over 90% of total milk produced from sheep and goats is used for dairy products. Despite these, the output of research performed in Greece on sheep and goats has never been assessed and no relevant studies have been published.

The present study is a scientometrics evaluation of published articles performed in Greece on sheep and goats during the last 25 years, a period that coincides with the implementation of reforms to shape and consolidate tertiary education establishments and research institutes in the country. The objectives of the study were the evaluation of the relevant publications and the presentation of quantitative characteristics regarding their scientific content and bibliometric details.

## 2. Materials and Methods

### 2.1. Search Procedure

The Web of Science platform (www.webofknowledge.com; accessed on 16 July 2022 Clarivate Analytics, Philadelphia, USA) was used for the search of relevant publications. The Web of Science Core Collection was employed; this includes the Science Citation Index Expanded, the Social Sciences Citation Index, the Arts and Humanities Citation Index, the Emerging Sources Citation Index, the Book Citation Index and the Conference Proceedings Citation Index, hence, the search spanned across multiple disciplines.

A topic search using the terms [[*sheep* OR goat*] OR [*Ovis aries* OR *Capra hircus*]] was performed (the asterisk served as a truncation symbol to include variations of the term (*goat* or *goats*). A topic search retrieved records that included the query terms in the title, the abstract or the keywords. The search was performed on 16 July 2022 (‘freeze date’).

The initial search produced 182,310 records. Document analysis of these was performed and the following types of documents were excluded: ‘meeting abstracts’, ‘notes’, ‘editorial materials’, ‘letters’, ‘early access’, ‘book reviews’, ‘news items’, ‘corrections’, ‘poetry’, ‘book chapters’, ‘corrections, additions’, ‘reprints’, ‘fiction, creative prose’, ‘film reviews’, ‘theatre reviews’, ‘discussions’, ‘record reviews’, ‘biographical items’, ‘art exhibit reviews’, ‘items about an individual’, ‘retracted publications’, ‘music performance review’, ‘retractions’, ‘abstract of published items’, ‘excerpts’, ‘TV review’, ‘radio review videos’, ‘music score review’, ‘bibliographies’, ‘hardware reviews’ and ‘scripts’. After those exclusions, a total of 161,166 records remained for further analysis.

Document analysis was performed again, and records were classified by country (Table S1). Only records from ‘Greece’ were selected, which retained 1711 records for further assessment. Of these, records with publication date before 1 July 1997 and after 30 June 2022 were excluded, hence the entire period of assessment was 25 years precisely. Thus, 1518 records were retained for further assessment.

These papers were individually assessed. Papers not including work related to sheep or goats were excluded. Moreover, papers with no Greece-based first or last authors (as indicated in the respective affiliations) were also excluded.

### 2.2. Paper Evaluation

After the above, 1080 records were retained for detailed assessment. In each paper, the following details were recorded.

- Relevant animal species (sheep, goats or both).
- Year of publication.
- Scientific establishment of origin (the establishment(s) of the first and last authors were taken into account).
- Journal in which published.
- General topic(s) of study in the paper: (i) production systems and sustainability, (ii) genetics and breeding, (iii) physiology, (iv) nutrition [(iva) applied nutrition, (ivb) nutritional biology and nutritional physiology, (ivc) feed technology], (v) animal products [(va) milk and dairy products, (vb) meat and meat products, (vc) wool and pelts, (vd) other], (vi) health and welfare [(via) diseases, (vib) health management and (vic) welfare], (vii) human-related models and (viii) society and professionals. A subsequent search was performed using the term [*welfare*] among the papers previously detected.
- Involvement of international collaboration (i.e., presence of co-authors with an affiliation in a country other than Greece).
- Total number of citations received.
- Number and names of all co-authors.
- Accessibility, i.e., whether there was open or subscription-only access to the paper.

### 2.3. Data Management and Analysis

For assessment of the impact of papers published, the following bibliometrics measures were employed: total number of citations received, *h*-index, *i_10_*-index. Moreover, the number of citations received by papers were also normalised by calculating the average citations per year after publication of each paper.

All data were entered into Microsoft Excel. Descriptive analysis was performed initially. The frequency of the various outcomes was evaluated in tables of cross-categorised frequency data by use of Pearson chi-square test as appropriate. Comparisons of proportions were performed by a two-proportion z-test. Comparisons between continuous data were performed by use of one-way analysis of variance. Pearson’s correlations were performed as indicated and significance of the result was evaluated according to the critical values for Pearson’s *r* [13]. Linear regression analysis was used to establish associations with the year of publication of each paper. Statistical significance was defined at *p* < 0.05.

## 3. Results

All the 1080 papers individually assessed, were indexed in the Web of Science, fulfilled the search criteria, originated from establishments based in Greece and presented work on sheep or goats. In total, 649 papers presented work solely on sheep, 197 papers presented work solely on goats and 234 papers presented work on both sheep and goats.

### 3.1. Year of Publication and Scientific Establishment of Origin of Papers

**Figure 1:**
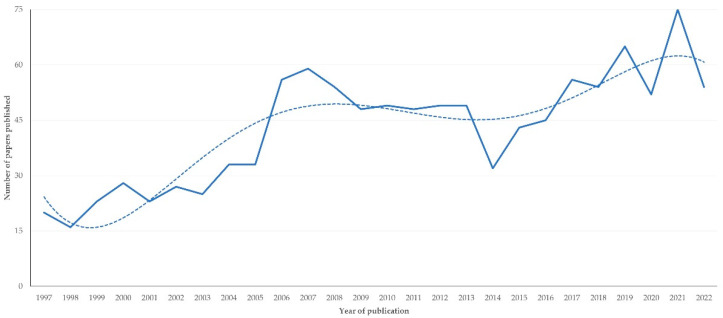
Number of papers on sheep and goats published annually during 1997–2022 from Greece (dashed line indicates trendline).

Throughout the study period, there was a clear progressive increase in numbers of papers published (slope ± standard error of the slope: 1.62 ± 0.24; *p* < 0.0001) (Figure 1).

Papers originated from 38 different entities in the country (Table S2). Most papers originated from universities (*n* = 984, 91.1%) and fewer ones from research establishments (*n* = 176, 16.3%), central government structures (*n* = 34, 3.1%), private sector companies (*n* = 8, 0.7%) or local authorities (*n* = 1, 0.1%). With regard to universities, most papers originated from the Aristotle University of Thessaloniki (*n* = 367, 34.0%), the University of Thessaly (*n* = 303, 28.0%) and the Agricultural University of Athens (*n* = 229, 21.2%); with regard to research establishments, most papers originated from the Hellenic Agricultural Organization—Dimitra (*n* = 147, 13.6%). Cumulatively, from these four establishments, originated 87.6% (*n* = 946) of all papers.

There was a significant difference (*p* = 0.019) in the origin of papers according to animal species, as some establishments published proportionately more papers on sheep than on goats (e.g., University of Thessaly: 31.4% versus 16.0% of all relevant papers), whilst others published more papers on goats than on sheep (e.g., Agricultural University of Athens: 22.7% versus 19.8%, and Hellenic Agricultural Organization—Dimitra: 16.2% versus 13.3% of all relevant papers). Details are in Table S2. There were also significant differences in the progressive change in the number of published papers between the above four establishments throughout the study period (Figure S1).

Within the Aristotle University of Thessaloniki, most papers were published by the Faculty of Veterinary Medicine (*n* = 268, 73.0% of all relevant papers published by the establishment and 24.8% of all relevant papers published in the country) and fewer ones by the Faculty of Agriculture (which, in Greece, also incorporates the field of food science) (*n* = 56, 15.3% of all papers relevant published by the establishment). Within the University of Thessaly, most papers were also published by the Veterinary Faculty (*n* = 237, 78.2% of all relevant papers published by the establishment and 21.9% of all relevant papers published in the country) and fewer ones by the Faculty of Medicine (*n* = 33, 10.9% of all relevant papers published by the establishment). Cumulatively, 465 papers (43.1% of all relevant papers published in the country) originated from the two veterinary faculties of the country, with fewer ones from faculties of agriculture (*n* = 359 papers, 33.2%) and faculties of medicine (*n* = 82 papers, 7.6%). At department level, most papers originated from the Department of Obstetrics and Reproduction of the Veterinary Faculty, University of Thessaly (*n* = 155 papers, 14.4% of all relevant papers published in the country) (Table S3 and Figure S2).

### 3.2. Journals in Which the Papers Were Published

In total, the 1080 papers were published in 318 different scientific journals. The journals in which most papers were published, were *Small Ruminant Research* (*n* = 166 papers) and *Journal of the Hellenic Veterinary Medical Society* (*n* = 41 papers). The journals (*n* = 19) in which at least 10 papers (*n* = 502 in total, 46.5% of all papers) have been published, are listed in Table 1.

**Figure 2:**
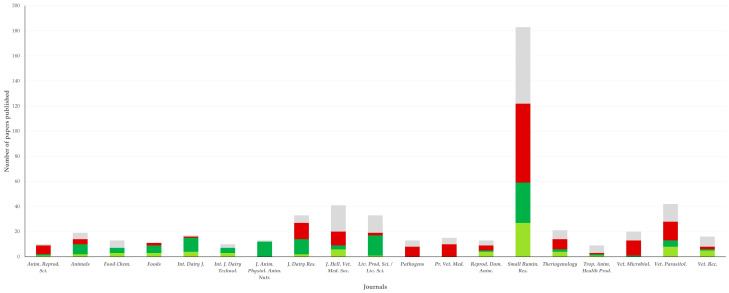
Association between journals (in italics at horizontal axis; standard abbreviations were used for journal titles) and establishments of origin of papers on sheep or goats published during 1997–2022 from Greece (grey: Aristotle University of Thessaloniki, burgundy: University of Thessaly, green: Agricultural University of Athens, green-yellow: Hellenic Agricultural Organization—Dimitra).

In total, 471 of the 502 papers in these 19 journals (93.8%) originated from one of the four establishments with the most papers published (Figure 2 and Figure S3). This proportion was significantly higher than the proportion of papers published from these four establishments in the other 299 journals (82.2%) (*p* < 0.0001).

### 3.3. General Topic of Study in the Papers

The most frequent general topic of study in the papers was health and welfare, which was covered in 504 (46.7%) papers. Second most frequent general topic was animal products (201 papers, 18.6%) (Table 2). There were some differences in the general topic of papers in accord with the animal species; for example, there was a significantly higher proportion of papers on physiology for sheep (11.9% of papers for sheep versus 5.3% of papers for goats; *p* < 0.0001) and of papers on animal products for goats (32.5% of papers for goats versus 15.3% of papers for sheep; *p* < 0.0001). Details are in Table S4.

**Figure 3:**
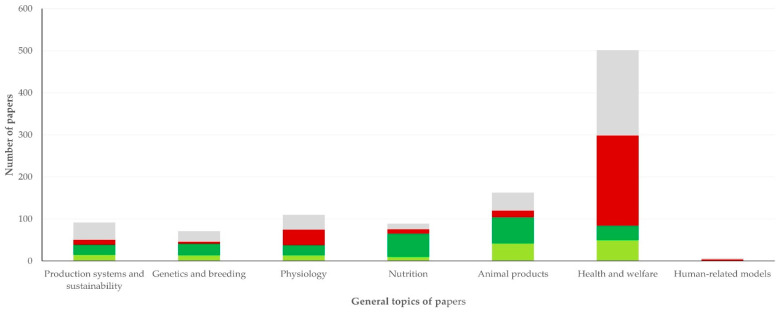
Stack-bar chart of the relative frequency of the general topics in papers on sheep and goats published during 1997–2022 from Greece, with origin from Aristotle University of Thessaloniki, University of Thessaly, Agricultural University of Athens and Hellenic Agricultural Organization—Dimitra (grey: Aristotle University of Thessaloniki, burgundy: University of Thessaly, green: Agricultural University of Athens, green-yellow: Hellenic Agricultural Organization—Dimitra).

There were also differences in the general topic of papers published by each of the above four establishments (*p* < 0.0001), as papers from the Aristotle University of Thessaloniki and the University of Thessaly focused on health and welfare of sheep and goats (*n* = 203, 55.3% of papers and *n* = 215, 71.0% of papers, respectively for each of the two universities), whilst papers from the Agricultural University of Athens focused on nutrition and animal products (*n* = 117, 51.1%). In general, however, output from the four main establishments referred to over 80% of all papers in six of the eight general topics (in genetics and breeding: 70.0% of all papers published on the topic, in human-related models: 33.3% of all papers published on the topic) (Figure 3 and Figure S4).

In seven of the general topics, most papers were published in *Small Ruminant Research*; differences were seen for the second and third most popular journal across the topics, although *Livestock Production Science* / *Livestock Science*, and to a lesser degree *Sustainability*, frequently published papers in a variety of topics (Table 3).

A total of 26 papers were found specifically in the field of welfare (i.e., with the respective search term). There was a clear progressive increase in the number of papers published annually: of the 26 papers, 4 (15.4%) were published before 2011 (i.e., on average, 0.4 paper annually), 15 (57.7%) were published from 2011 to 2020 (i.e., on average, 1.5 papers annually) and 6 (23.1%) were published in 2021 and 2022 (i.e., on average, 4.0 papers annually) (slope ± standard error of the slope: 0.14 ± 0.03; *p* < 0.0001) (Figure S5).

With regard to specific diseases, most of the relevant 403 papers dealt with diseases of multi-system topography (*n* = 93) or diseases of the udder (*n* = 79). Most diseases described in the papers were of bacterial (*n* = 164) or endo-parasitic (*n* = 81) nature (Table S5).

### 3.4. Involvement of International Collaboration

Involvement of international collaboration was identified in 299 papers (27.7%) (27.5% of papers on sheep and 30.1% on those on goats, *p* = 0.15). International co-authors were affiliated with establishments in 38 countries, more frequently in the United Kingdom (*n* = 89 papers), France (*n* = 38) and Italy (*n* = 36) (Table S6).

Involvement of international collaborations was more frequent among the four establishments with the most published papers than among other ones: 27.0% versus 11.7% of papers with origin from respective establishments (*p* < 0.0001).

With regard to the general topic of study, papers on health and welfare (33.7% of papers on that topic) and on human-related models (33.3% of papers on that topic) had more frequently involvement of international collaborations than papers on other topics (*p* = 0.001 for differences between the topics; Table S7).

### 3.5. Impact of Papers

In total, the 1080 papers have received 16,558 citations, i.e., οn average (± standard error of the mean) 15.4 ± 0.6 citations per paper; overall, the *h*-index was 56, the *i_10_*-index was 518 and the yearly citations per paper were 1.71 ± 0.06. Generally, papers on goats had higher impact measures than papers on sheep: 16.6 ± 1.1 and 14.8 ± 0.8 (*p* =0.06) citations per paper, 44 and 49 *h*-index (*p* = 0.002), 206 and 406 *i_10_*-index (*p* = 0.63) and 1.79 ± 0.09 and 1.51 ± 0.06 yearly citations per paper (*p* =0.018), respectively.

There were no significant differences between the citations received by papers with origin the four establishments with the higher number of papers (Table S8). There was however some tendency for higher impact measures in papers with involvement of international collaboration (Table 4).

**Figure 4:**
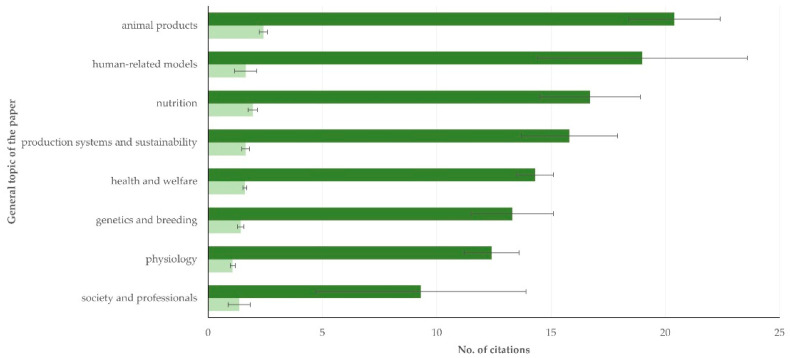
Mean no. of citations (dark green: average total number, light green: average yearly number) received by papers on sheep and goats published during 1997–2022 from Greece, in accord with the general topic of the papers (bars indicate standard error of the mean).

Papers on animal products and on human-related models were the ones with the most citations: 20.4 ± 2.0 and 19.0 ± 4.6 average citations, respectively, and 2.42 ± 0.18 and 1.65 ± 0.49 average yearly citations, respectively. The differences between the various general topics of the papers (Figure 4) were significant (*p* = 0.006 for differences in average citations and *p* < 0.0001 for differences in average yearly citations).

### 3.6. Authors of Papers

Cumulatively, in the 1080 papers, there were 5770 co-authors, i.e., on average 5.3 ± 0.1 co-authors per paper (median: 5, min-max: 1–21). In total, there were 1711 individual authors of the papers; among these, 25 authors have co-authored at least 27 papers each (i.e., ≥ 2.5% of total number of papers published), of which 7 authors at least 54 papers each (i.e., ≥ 5.0% of total number of papers published); one author has co-authored 162 papers (i.e., 15.0% of total papers published). Of these 25 authors, 11 were affiliated with University of Thessaly, 9 with Artistotle University of Thessaloniki, 3 with Agricultural University of Athens and one with Hellenic Agricultural Organization—Dimitra.

Cumulatively, these 25 authors were first or last authors in 53.1%, 36.8%, 28.4% and 6.1% of all papers published by the establishments with which they were affiliated, respectively.

**Figure 5:**
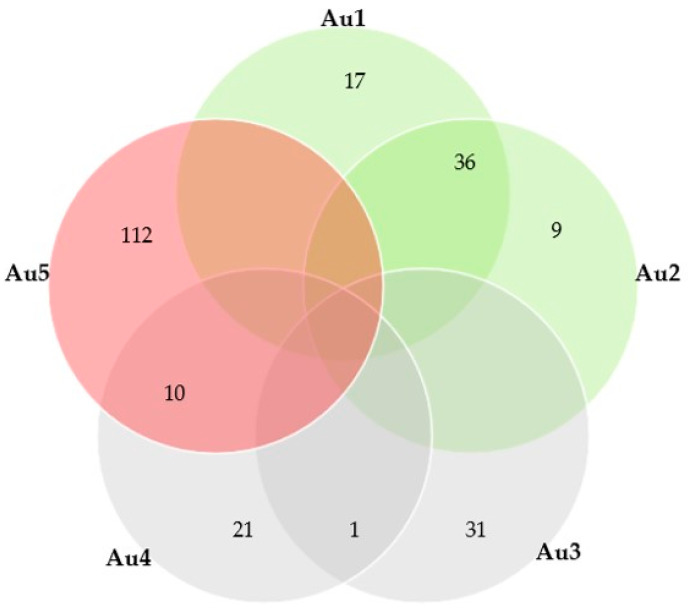
Venn diagram of numbers of papers published by each of the five individuals with at least 27 papers each as first or last author on their own and of numbers of the respective collaborative papers (grey shapes: authors affiliated with Aristotle University of Thessaloniki, burgundy shape: author affiliated with University of Thessaly, green shapes: authors affiliated with Agricultural University of Athens; Au1-5: descriptors for the five authors, with characterising numbers in random order).

Of the above authors, 728 individuals were first or last authors in the papers; among these, 5 authors were first or last in at least 27 papers each (max: 122 papers). Cumulatively, these 5 individuals were first or last authors in 237 papers (22.0% of all published papers). There appeared to be limited interaction and collaboration between them in publishing papers jointly (Figure 5).

**Figure 6:**
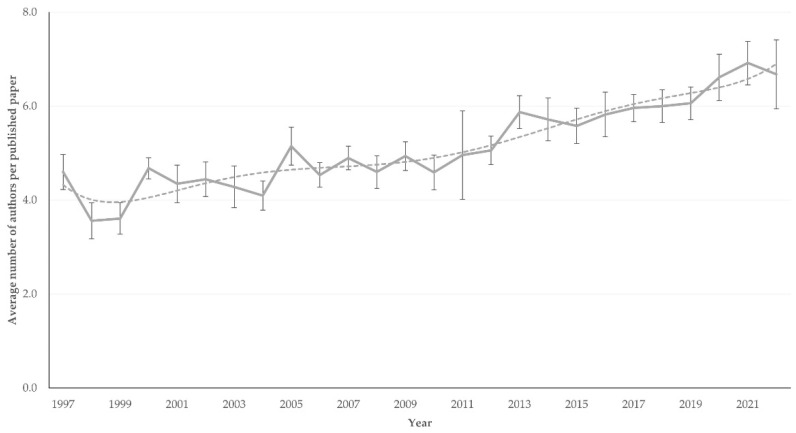
Average number of co-authors in papers on sheep and goats published annually during 1997–2022 from Greece (dashed line indicates trendline).

The average (± standard error of the mean) number of co-authors per paper progressively increased from 4.6 ± 0.4 and 3.6 ± 0.4 in 1997 and 1998, respectively, to 6.9 ± 0.5 and 6.7 ± 0.7 in 2021 and 2022, respectively (slope ± standard error of the slope: 0.10 ± 0.01; *p* < 0.0001) (Figure 6). In 36 papers (3.3%), there was only one author.

The average number of authors in papers with origin in any of the four establishments with largest number of papers did not differ significantly to that in papers with origin in any of the other establishments: 5.3 ± 0.1 versus 5.4 ± 0.1 (*p* = 0.42). Moreover, there was a mild correlation between the number of authors in a paper and yearly citations per paper (*r* = 0.1027, *p* = 0.0004). Finally, there was a clear difference in the number of authors of papers in accord to the general topic of the paper (*p* < 0.0001) (Table 5).

### 3.7. Accessibility of Papers

**Figure 7:**
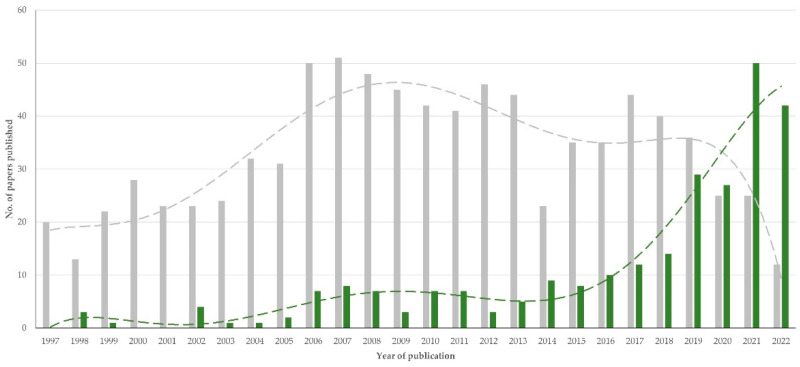
Number of papers on sheep and goats published annually during 1997–2022 in Greece with open (green) or subscription-only (grey) access (dashed lines indicate respective trendlines).

In total, 237 (21.9%) papers were published with open access. There was clear evidence that, after 2019, papers published with open access outnumbered those published with subscription-only access (Figure 7). Respective slopes (± standard error of slope) were 1.30 ± 0.22 versus 0.31 ± 0.30 (*p* = 0.011). Proportionately, more papers in the general topics of society and professionals (37.5% of those in the topic) and genetics and breeding (35.0% of those in the topic) were published with open access than papers in other topics (*p* = 0.0001) (Table S9). Papers published with open access received significantly more citations than those published as subscription-only: 2.07 ± 0.15 versus 1.41 ± 0.07 yearly citations per paper (*p* = 0.003).

## 4. Discussion

### 4.1. Research Output on Sheep and Goats from Greece

Small ruminant farming is currently the most important animal farming business in Greece and generates approximately 18% of the total primary sector income [8]. This is larger than respective proportion in the gross domestic product of other countries. For example, a comparison with Spain, another country of South Europe with high small ruminant numbers (16.0 M sheep and 2.8 M goats [9]), reveals that the sheep and goat industry in that country contributes with only 3% to the total agricultural income of the country [14]. However, research output from Spain was larger: 7524 records from Spain versus 1706 records from Greece, were identified during the initial analysis of documents. We postulate that this difference can be due, at least to some extent, to differences in research funding between the two countries: for example, from 1997 to 2018, Greece has been allocating 0.43% to 1.21% of annual state budget to research activities, whilst Spain has been allocating 0.79% to 1.36% of it [15]. Moreover, the relevant establishments available in the countries may also play a role: there are two veterinary and two animal science faculties in Greece and 11 and 15 faculties, respectively, in Spain.

A comparison with Turkey, another country in the area with high sheep and goat populations (42.0 M sheep and 11.9 M goats [16]), reveals the same result. Although the sheep and goat industry in that country is estimated to contribute with <10% to the total agricultural income of the country [17], research output was still higher: 4091 records from Turkey versus 1706 records from Greece, were identified during the initial analysis of documents. Again, the country has a larger number of academic establishments than Greece, specifically, 11 veterinary and over 20 animal science faculties.

The above points indicate that possibly the size of a country (which is associated with financial measures, e.g., gross domestic product and consequently, among others, funding of research) and the extent of its educational system (which is also related to the size and the population of that country) seem to be more also important for the total research output additionally to the relative importance of the livestock sector within the country.

Nevertheless, research output from Greece has increased progressively. There was a stability in the number of papers published during 2011 to 2015, which coincided with the financial problems of the country, a consequence of which was the significant reduction in funding of public activities, including research. Increase has resumed after 2015 and again a steady growth has been established.

### 4.2. Establishments and Authors

The findings indicate that research work on sheep and goats and consequent output have centred around four establishments in the country. These are the two veterinary faculties of the country, the Agricultural University of Athens (which covers widely topics related to animal production and food production) and an agricultural research institute.

These establishments have attracted more frequently international collaborations, as they can be ‘seen’ more often in the international scene. International collaboration, which is an important branch of bibliometric indicators [18], can provide access to more researchers with a variety of skills and knowledge; also, international collaborations can contribute to a wider reach of published works. This was confirmed in the present study, where it was found that papers with international collaborations made a higher impact.

Five persons affiliated with the above establishments have authored (as first or last authors) most papers. Moreover, most authors with a large number of papers were affiliated with these establishments, which indicates the presence of large groups active in the topic within these institutions. However, few collaborative inter-establishment papers were found. Possibly, international collaborations might be preferred over work with other establishments within the country. Differences in research interests (as shown on the analysis of the topics of papers according to establishment) or rules for funding projects may also potentially account for this limited national collaboration. However, science and research can provide solutions to various problems, and thus collaborations need to be established at various levels, nationally and internationally [19].

### 4.3. Topics of Research

Reasonably, in the country, research on sheep and goats is focused on dairy production. Most papers on the general topic of animal products have dealt with dairy products technology and safety, whilst only few papers have dealt with meat products. Further, Greece has already been identified to have a high output on sheep mastitis [15]. Those findings have been corroborated in the present analysis, which showed that udder problems were the small ruminant disease, on which most research has been performed in the country.

The present findings are aligned with the production systems prevailing in the country. Nevertheless, further work needs to be performed on meat production and this is an area identified for future studies. In this respect, the results of scientometrics studies could be of use and could be considered by governments, grant-giving bodies, and researchers to direct resources of research and perform relevant studies accordingly.

### 4.4. Journals and Impact

Most papers have been published on *Small Ruminant Research*, an international journal with a thematic approach to sheep and goat research. Such journals are favoured by researchers, as they address their work to a specialised audience with greater interest in relevant studies. Moreover, scientists working on a topic possibly read systematically thematic journals, thus become aware more easily about developments in research in their work area. The *Journal of the Hellenic Veterinary Medical Society* is a journal published by a national society; the journal, further to the international audience, is also widely distributed to local veterinarians, members of the Society; hence, authors can use publications in that journal as a means to inform the national community about matters of interest. The journal has been published continuously since 1950 and has been listed in the Web of Science since 2010, after confirmation that it met the inclusion criteria.

The sharp increase in numbers of articles published in open access is compatible with the increasing support of the European Commission for this mode of publication and the requirement for using this approach for all publications derived from EU-funded research [20]. Reasonably, papers published as open access had received more citations than ones published as subscription-only, because, as they are freely available, they can be read and, thus cited, more frequently and by more people.

### 4.5. Implications

Scientometrics takes into account objective measurements and indexes to assess independently and objectively scientific research. First, the results can be used to better orientate relevant research, e.g., by detecting little-studied topics, by identifying researchers with relevant outcomes, by classifying establishments, ultimately by exploring the broader impact of research.

Moreover, the results can be used in the evaluation of research performance and research outputs by research groups and academic establishments of various levels for many purposes, e.g., assessment or resource allocation. Certainly, the results of scientometrics studies are not meant to replace peer-reviewed assessments. However, these evaluations can be supported by scientometrics results, so-termed ‘informed per-review’ [21], and can be used to facilitate, not replace, assessors.

## 5. Conclusions

The findings have indicated focusing of research on sheep and goats in Greece in areas associated with dairy production. It is noted that research in meat production in all its facets (e.g., meat technology, diseases of lambs and kids) will also be of benefit for the country and should be instituted to support this part of production from small ruminant farms. Such research can also attract further international collaborations and funding. Moreover, the findings have indicated accumulation of research on sheep and goats in Greece in only some establishments. These institutions and the respective researchers have accumulated expertise on this scientific area and have been contributing on it by expanding their work. The presence of large groups in these establishments favours scientific output. The findings of the study can be employed to initiate improved relevant research approaches in the country.

## Figures and Tables

**Table 1:** List of journals in which most papers on sheep and goats were published during 1997–2022 from Greece.

Title of Journal	No. of Papers Published (Percentage of All Papers)
*Small Ruminant Research*	166 (15.4)
*Journal of the Hellenic Veterinary Medical Society*	41 (3.8)
*Veterinary Parasitology*	36 (3.3)
*Livestock Production Science* / *Livestock Science*	32 (3.0)
*Journal of Dairy Research*	30 (2.8)
*Veterinary Microbiology*	23 (2.1)
*Animals*	19 (1.8)
*International Dairy Journal*	18 (1.7)
*Theriogenology*	18 (1.7)
*Food Chemistry*	15 (1.4)
*Journal of Animal Physiology and Animal Nutrition*	14 (1.3)
*Foods*	13 (1.2)
*Veterinary Record*	13 (1.2)
*International Journal of Dairy Technology*	11 (1.0)
*Pathogens*	11 (1.0)
*Preventive Veterinary Medicine*	11 (1.0)
*Reproduction in Domestic Animals*	11 (1.0)
*Animal Reproduction Science*	10 (0.9)
*Tropical Animal Health and Production*	10 (0.9)

**Table 2:** General topic of study in papers on sheep or goats published during 1997 to 2022 from Greece.

General Topic of Study in Published Papers	No. of Papers Published (Percentage)
Health and welfare	504 (46.7)
diseases	403 (80.0)
health management	95 (18.8)
welfare	6 (1.2)
Animal products	201 (18.6)
milk and dairy products	186 (92.5)
meat and meat products	17 (8.5)
wool and pelts	0 (0.0)
other	0 (0.0)
Physiology	125 (11.6)
Production systems and sustainability	113 (10.5)
Nutrition	101 (9.4)
applied nutrition	74 (73.3)
nutritional biology & nutritional physiology	25 (24.8)
feed technology	2 (2.0)
Genetics and breeding	100 (9.3)
Human-related models	21 (1.9)
Society and professionals	16 (1.5)

**Table 3:** List of journals in which most papers on sheep and goats were published during 1997 to 2022 from Greece, according to general topic of the paper (standard abbreviations were used for journal titles).

General Topic of Study in Published Papers	Journal in Which Most Papers Were Published (Percentage of Papers in the General Topic)		
	1^st^	2^nd^	3^rd^
Health and welfare	*Small Rumin. Res.* (16.7)	*Vet. Parasitol.* (7.2)	*J. Hell. Vet. Med. Soc.* (5.4)
Animal products	*Small Rumin. Res.* (9.1)	*Int. Dairy Technol. J.* (7.7)	*Food Chem.* (7.2)
Physiology	*Small Rumin. Res.* (14.9)	*Theriogenology* (9.9)	*Reprod. Dom. Anim.* (5.8)
Production systems and sustainability	*Small Rumin. Res.* (15.9)	*Liv. Prod. Sci.*/*Liv. Sci.* (11.5)	*Sustainability* (5.3)
Nutrition	*Small Rumin. Res.* (19.2)	*J. Anim. Physiol. Anim. Nutr.* (12.1)	*Liv. Prod. Sci.*/*Liv. Sci.* (9.1)
Genetics and breeding	*Small Rumin. Res.* (18.1)	*Liv. Prod. Sci.*/*Liv. Sci.* & *Plos One* (7.4)	
Human-related models	21 different journals each with one paper		
Society and professionals	*Small Rumin. Res.* (25.0)	*Sustainability* (12.5)	10 different journalseach with one paper

**Table 4:** Measures of impact of papers on sheep and goats published during 1997–2022 from Greece, according to involvement of international collaboration.

	Total Citations	Mean Citations Per Paper (± Standard Error of the Mean)	*h*-Index	*i_10_*-Index	Average Yearly Citations Per Paper
Papers with international collaboration (*n* = 298)	4971	16.7 ± 1.3	34	154	2.04 ± 0.13
Papers without international collaboration (*n* = 782)	11,558	14.9 ± 0.7	49	364	1.59 ± 0.07
*p* value		0.20	0.005	0.15	0.002

**Table 5:** Average number of co-authors in papers on sheep and goats published during 1997–2022 from Greece, according to general topic of the paper.

General Topic of Study in Published Papers	Average Number of Co-authors (± Standard Error of the Mean)
Human-related models	6.4 ± 0.5
Health and welfare	5.9 ± 0.1
Physiology	5.2 ± 0.2
Genetics and breeding	5.1 ± 0.2
Animal products	4.8 ± 0.2
Nutrition	4.7 ± 0.2
Production systems and sustainability	4.4 ± 0.2
Society and professionals	3.5 ± 0.4

## Data Availability

All data are available in the Web of Science platform (www.webofknowledge.com, accessed on 1 October 2022).

## Supplementary Materials

The following supporting information can be downloaded at: https://www.mdpi.com/article/10.3390/ani12192666/s1. Figure S1: Trends for annual numbers of published papers on sheep and goats during 1997–2022 for Aristotle University of Thessaloniki, University of Thessaly, Agricultural University of Athens and Hellenic Agricultural Organization—Dimitra; Figure S2: Academic departments with highest number of papers on sheep and goats published from 1997–2022 in Greece; Figure S3: Association between journals and establishments of origin of papers on sheep or goats published during 1997–2022 in Greece; Figure S4: Ring-pie of the relative frequency of the general topics in papers on sheep and goats published during 1997–2022, with origin from Aristotle University of Thessaloniki, University of Thessaly, Agricultural University of Athens and Hellenic Agricultural Organization—Dimitra; Figure S5: Number of papers on welfare of sheep and goats published annually from Greece during 1997–2022; Table S1: Analysis of documents (*n* = 161,166 records) found in Web of Science after search using the terms [[*sheep* OR *goat**] OR [*Ovis aries* OR *Capra hircus*]], according to the country of origin; Table S2: Establishments of origin of 1080 papers on sheep and goats published during 1997–2022 in Greece; Table S3: Academic departments with highest number of papers on sheep and goats published from 1997–2022 in Greece; Table S4: General topic of study in papers on sheep and goats published during 1997 to 2022 in Greece; Table S5: Details of papers on sheep and goats in papers published during 1997 to 2022 in Greece, according to the topography and the nature of diseases described in the papers (in alphabetical order); Table S6: Countries with establishments with which there was international collaboration in papers on sheep or goats published during 1997 to 2022 in Greece, according to the continent and the country of origin (in alphabetical order); Table S7: Papers on sheep and goats published during 1997 to 2022 in Greece, in which there was international collaboration, according to the general topic of the study; Table S8: Measures of impact of papers on sheep and goats published during 1997–2022 in Greece, with origin from the four establishments with most papers; Table S9: Proportion of papers on sheep and goats published during 1997 to 2022 in Greece with open access, according to the general topic of the study.
