# Supplementary material for: Scientometrics Study of Research Output on Sheep and Goats from Greece
Source: Animals (Basel). 2022 Oct 4;12(19):2666. doi: 10.3390/ani12192666 (PMC9559483; doi:10.3390/ani12192666)
Supplement: Supplementary file 1 [file animals-12-02666-s001.zip › animals-1901544-supplementary.pdf]

Scientometrics Study of Research Output on Sheep and Goats from Greece

Daphne T. Lianou and George C. Fthenakis

Table S1. Analysis of documents (*n* = 161,166 records) found in Web of Science after search using the terms [[*sheep* OR *goat**] OR [*Ovis aries* OR *Capra hircus*]][1], according to the country of origin (in alphabetical order).

Country	No. of records found	Percentage
Afghanistan	21	0.01
Albania	38	0.02
Algeria	362	0.22
Andorra	2	0.00
Angola	10	0.01
Anguilla	1	0.00
Antigua	2	0.00
Argentina	1626	1.01
Armenia	20	0.01
Australia	13,738	8.52
Austria	12546	7.78
Azerbaijan	19	0.01
Bahamas	2	0.00
Bahrain	4	0.00
Bangladesh	322	0.20
Barbados	4	0.00
Belarus	24	0.01
Belgium	2159	1.34
Benin	52	0.03
Bermuda	1	0.00
Bhutan	33	0.02
Bolivia	47	0.03
Bosnia-Herzegovina	60	0.04
Botswana	73	0.05
Brazil	7708	4.78
Brunei	3	0.00
Bulgaria	337	0.21
Burkina Faso	118	0.07
Burundi	12	0.01
Cambodia	12	0.01

Cameroon	102	0.06
Canada	5793	3.59
Cape Verde	2	0.00
Central African Republic	5	0.00
Chad	24	0.01
Chile	680	0.42
Colombia	248	0.15
Comoros	4	0.00
Costa Rica	68	0.04
Cote Ivoire	65	0.04
Croatia	487	0.30
Cuba	140	0.09
Curacao	1	0.00
Cyprus	173	0.11
Czech Republic	1348	0.84
Democratic Republic of Congo	35	0.02
Denmark	1358	0.84
Djibouti	6	0.00
Dominica	4	0.00
Dominican Republic	4	0.00
Ecuador	119	0.07
Egypt	1769	1.10
El-Salvador	4	0.00
Equatorial Guinea	1	0.00
Eritrea	17	0.01
Estonia	88	0.05
Eswatini	8	0.00
Ethiopia	1025	0.64
Fiji	17	0.01
Finland	722	0.45
France	8741	5.42
Gabon	18	0.01
Gambia	50	0.03
Georgia	21	0.01
Germany	8439	5.24
Ghana	170	0.11
Greece	1706	1.06
Grenada	13	0.01
Guatemala	4	0.00
Guinea	11	0.01
Guinea-Bissau	1	0.00
Haiti	3	0.00
Honduras	5	0.00

Hong-Kong	24	0.01
Hungary	753	0.47
Iceland	180	0.11
India	9265	5.75
Indonesia	464	0.29
Iran	3280	2.04
Iraq	382	0.24
Ireland	1092	0.68
Israel	1208	0.75
Italy	6187	3.84
Jamaica	10	0.01
Japan	4774	2.96
Jordan	454	0.28
Kazakhstan	147	0.09
Kenya	951	0.59
Kosovo	19	0.01
Kuwait	95	0.06
Kyrgyzstan	46	0.03
Laos	28	0.02
Latvia	24	0.01
Lebanon	104	0.06
Lesotho	22	0.01
Liberia	2	0.00
Libya	65	0.04
Liechtenstein	1	0.00
Lithuania	96	0.06
Luxembourg	28	0.02
Macedonia	17	0.01
Madagascar	27	0.02
Malawi	28	0.02
Malaysia	750	0.47
Mali	43	0.03
Malta	11	0.01
Mauritania	27	0.02
Mauritius	9	0.01
Mexico	2429	1.51
Moldova	22	0.01
Monaco	3	0.00
Mongolia	119	0.07
Montenegro	14	0.01
Morocco	443	0.27
Mozambique	60	0.04
Myanmar	21	0.01

Namibia	59	0.04
Nepal	120	0.07
Netherlands	3504	2.17
New Zealand	5212	3.23
Nicaragua	7	0.00
Niger	57	0.04
Nigeria	1208	0.75
North Korea	1	0.00
North Macedonia	17	0.01
Norway	1853	1.15
Oman	140	0.09
Pakistan	1488	0.92
Palestine	23	0.01
Panama	21	0.01
Papua New Guinea	8	0.00
Paraguay	10	0.01
Peoples' Republic of China	10,360	6.43
Peru	183	0.11
Philippines	108	0.07
Poland	2476	1.54
Portugal	947	0.59
Qatar	33	0.02
Republic of Congo	7	0.00
Romania	445	0.28
Russia	1032	0.64
Rwanda	25	0.02
Samoa	17	0.01
Sao Tome & Principe	2	0.00
Saudi Arabia	958	0.59
Senegal	170	0.11
Serbia	296	0.18
Seychelles	3	0.00
Sierra Leone	7	0.00
Singapore	160	0.10
Slovakia	737	0.46
Slovenia	251	0.16
Somalia	13	0.01
South Africa	2607	1.62
South Korea	1030	0.64
South Sudan	6	0.00
Spain	7524	4.67
Sri Lanka	100	0.06
St Kitts & Nevi	66	0.04

Sudan	429	0.27
Sweden	2065	1.28
Switzerland	2758	1.71
Syria	173	0.11
Taiwan	415	0.26
Tajikistan	19	0.01
Tanzania	296	0.18
Thailand	506	0.31
Togo	17	0.01
Tonga	3	0.00
Trinidad and Tobago	66	0.04
Tunisia	530	0.33
Turkey	4091	2.54
Turkmenistan	2	0.00
United Arab Emirates	208	0.13
Uganda	162	0.10
Ukraine	108	0.07
United Kingdom	18,441	11.44
United States of America	33,232	20.62
Uruguay	706	0.44
Uzbekistan	35	0.02
Venezuela	240	0.15
Vietnam	154	0.10
Western Samoa	1	0.00
Yemen	35	0.02
Zambia	86	0.05
Zimbabwe	222	0.14

[1] Items in following categories were excluded: ‘meeting abstracts’, ‘notes’, ‘editorial materials’, ‘letters’, ‘early access’, ‘book reviews’, ‘news items’, ‘corrections’, ‘poetry’, ‘book chapters’, ‘corrections, additions’, ‘reprints’, ‘fiction, creative prose’, ‘film reviews’, ‘theatre reviews’, ‘discussions’, ‘record reviews’, ‘biographical items’, ‘art exhibit reviews’, ‘items about an individual’, ‘retracted publications’, ‘music performance review’, ‘retractions’, ‘abstract of published items’, ‘excerpts’, ‘TV review, radio review videos’, ‘music score review’, ‘bibliographies’, ‘hardware reviews’ and ‘scripts’.

Table S2. Establishments of origin of 1080 papers on sheep and goats published during 1997 – 2022 in Greece.

Establishment	All papers		Papers on sheep		Papers on goats	
	No. of papers	Percentage	No. of papers	Percentage	No. of papers	Percentage
Aristotle University of Thessaloniki	367	34.0	297	33.6	144	33.4
University of Thessaly	303	28.1	278	31.4	69	16.0
Agricultural University of Athens	229	21.2	175	19.8	98	22.7
Hellenic Agricultural Organization – Dimitra	147	13.6	117	13.3	70	16.2
University of Ioannina	38	3.5	26	2.9	16	3.7
Ministry of Agricultural Development and Food	27	2.5	24	2.7	8	1.9
National and Kapodistrian University of Athens	27	2.5	17	1.9	19	4.4
University of Patras	25	2.3	23	2.6	8	1.9
International University of Greece	23	2.1	18	2.0	10	2.3
Democritus University of Thrace	16	1.5	12	1.4	7	1.6
University of Crete	14	1.3	11	1.2	11	2.6
National Centre for Research and Technological Development	9	0.8	7	0.8	5	1.2
Private companies (various entities, *n* = 9)	9	0.8	8	0.9	5	1.2
Academy of Athens	7	0.6	6	0.7	3	0.7
University of the Aegean	7	0.6	7	0.8	1	0.2
University of Western Macedonia	6	0.6	6	0.7	2	0.5
Democritus Research Centre	5	0.5	2	0.2	4	0.9
University of West Attica	5	0.5	4	0.5	1	0.2
Ministry of Defence	4	0.4	4	0.5	2	0.5
Technical University of Athens	4	0.4	3	0.3	2	0.5
Foundation for Research and Technology	3	0.3	3	0.3	1	0.2
Ministry of Health	3	0.3	3	0.3	1	0.2
Panteion University	3	0.3	3	0.3	0	0.0
Pasteur Institute	3	0.3	3	0.3	0	0.0
Charokopion University	1	0.1	1	0.1	0	0.0
Hellenic Foundation for Research	1	0.1	1	0.1	1	0.2
Hellenic Mediterranean University	1	0.1	1	0.1	1	0.2
Local Authority of Larissa	1	0.1	1	0.1	1	0.2
University of Macedonia	1	0.1	1	0.1	1	0.2
University of Peloponnese	1	0.1	0	0.0	1	0.2

Figure S1. Trends for annual numbers of published papers on sheep and goats during 1997 – 2022 for Aristotle University of Thessaloniki (slope ± standard error of the slope: 0.158 ± 0.111), University of Thessaly (0.568 ± 0.132), Agricultural University of Athens (0.443 ± 0.101) and Hellenic Agricultural Organization – Dimitra (0.167 ± 0.053) (University of Thessaly compared to Aristotle University of Thessaloniki or Hellenic Agricultural Organization – Dimitra: $p < 0.022$; Agricultural University of Athens compared to Hellenic Agricultural Organization – Dimitra: $p = 0.019$; for all other comparisons: $p > 0.06$).

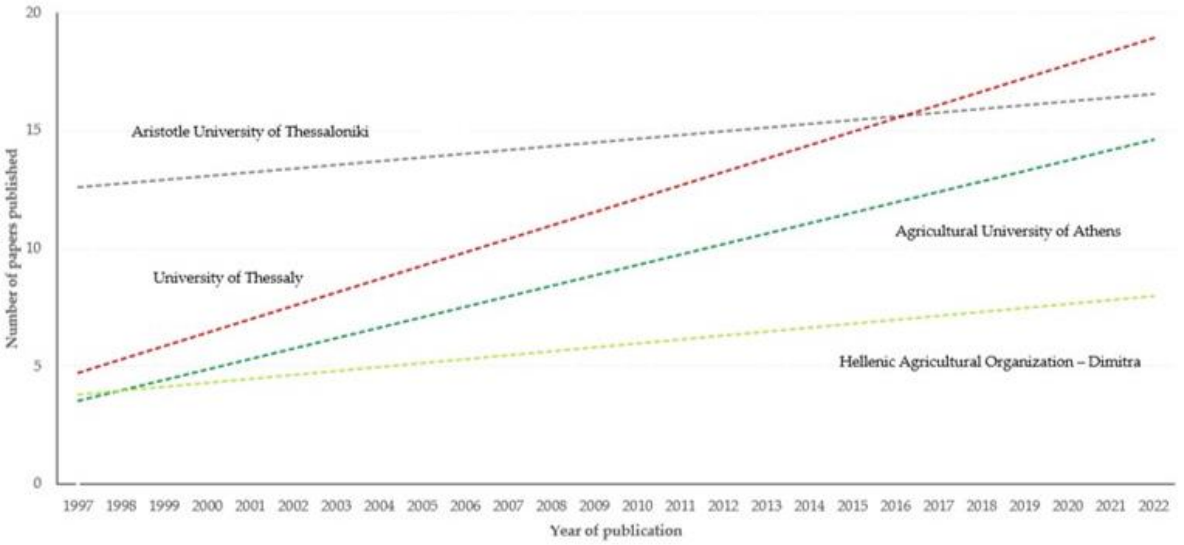

Table S3. Academic departments with highest number of papers on sheep and goats published from 1997 – 2022 in Greece.

General topic of study	No. of papers	Percentage
University of Thessaly, Veterinary Faculty, Department of Obstetrics and Reproduction	155	14.4
Aristotle University of Thessaloniki, Faculty of Veterinary Medicine, Farm Animal Clinic	83	7.7
Agricultural University of Athens, Faculty of Animal Science, Laboratory of Nutritional Physiology and Feeding	72	6.7
Agricultural University of Athens, Faculty of Animal Science, Laboratory of Animal Breeding and Husbandry	72	6.7
Aristotle University of Thessaloniki, Faculty of Veterinary Medicine, Laboratory of Animal Husbandry	54	5.0
Agricultural University of Athens, Faculty of Animal Science, Laboratory of Animal Anatomy and Physiology	36	3.3
Aristotle University of Thessaloniki, Faculty of Veterinary Medicine, Laboratory of Parasitology and Parasitic Diseases	36	3.3
Aristotle University of Thessaloniki, Faculty of Veterinary Medicine, Laboratory of Microbiology and Infectious Diseases	35	3.2
University of Thessaly, Veterinary Faculty, Department of Medicine	33	3.1
Agricultural University of Athens, Faculty of Food Science, Department of Dairy Science	30	2.8

Figure S2. Academic departments with highest number of papers on sheep and goats published from 1997 – 2022 in Greece (UTH: University of Thessaly, AUTH: Aristotle University of Thessaloniki, AUA: Agricultural University of Athens, F: Faculty, D: Department, C: Clinic, L: Laboratory)

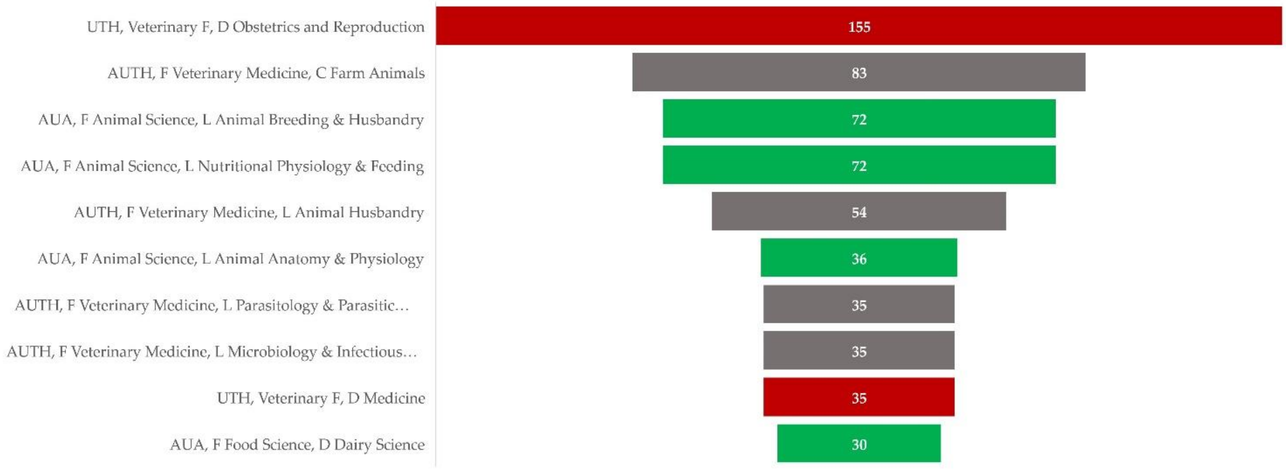

Figure S3. Association between journals (in italics at horizontal axis; standard abbreviations were used for journal titles) and establishments of origin of papers on sheep or goats published during 1997 – 2022 in Greece (gray bars: Aristotle University of Thessaloniki, burgundy bars: University of Thessaly, green bars: Agricultural University of Athens, green-yellow bars: Hellenic Agricultural Organization – Dimitra).

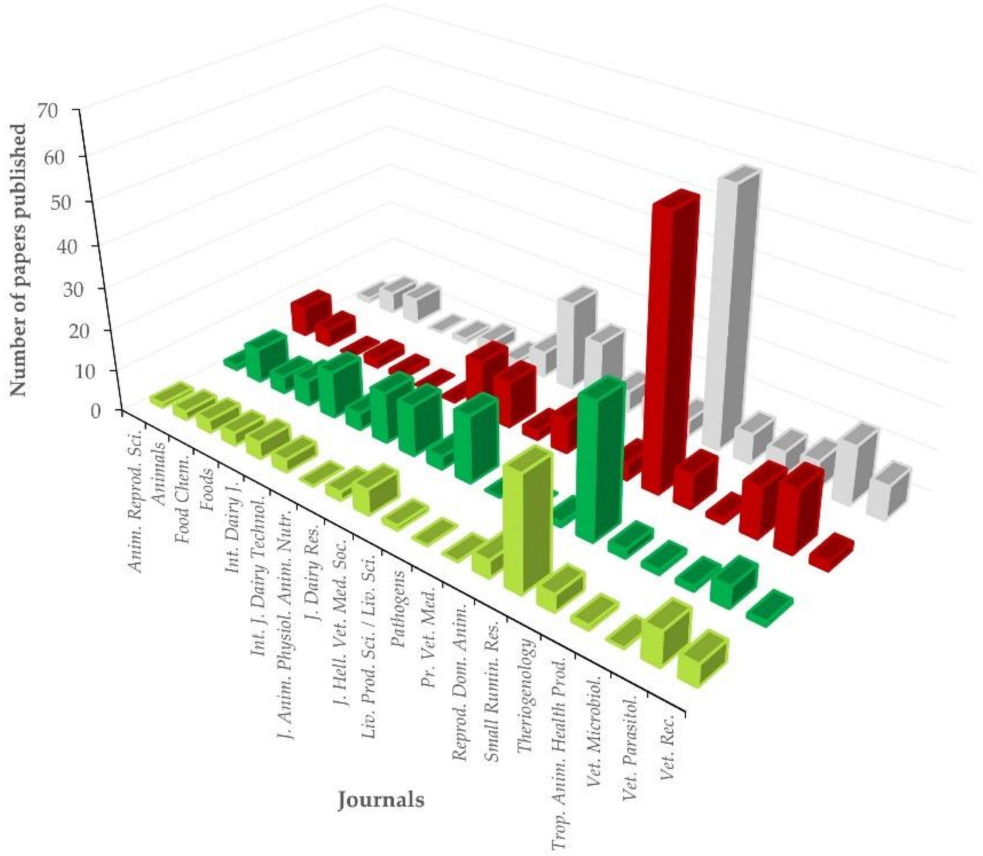

Table S4. General topic of study in papers on sheep and goats published during 1997 to 2022 in Greece.

General topic of study	Papers on sheep		Papers on goats	
	No. of papers	Percentage	No. of papers	Percentage
Health and welfare	442	50.1	178	41.3
Animal products	135	15.3	140	32.5
Physiology	105	11.9	23	5.3
Production systems and sustainability	96	10.9	45	10.4
Nutrition	73	8.3	38	8.8
Genetics and breeding	77	8.7	29	6.7
Human-related models	20	2.3	2	0.5
Society and professionals	12	1.4	12	2.8

Figure S4. Ring-pie of the relative frequency of the general topics in papers on sheep and goats published during 1997 – 2022, with origin from Aristotle University of Thessaloniki, University of Thessaly, Agricultural University of Athens and Hellenic Agricultural Organization – Dimitra (outer to inner ring, respectively).

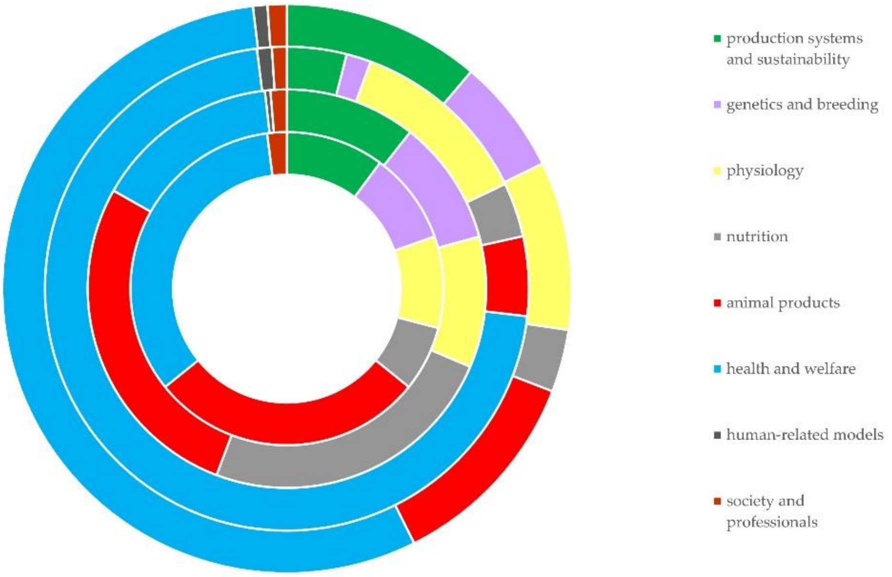

Figure S5. Number of papers on welfare of sheep and goats published annually from Greece during 1997 – 2022 (dashed line indicates trendline).

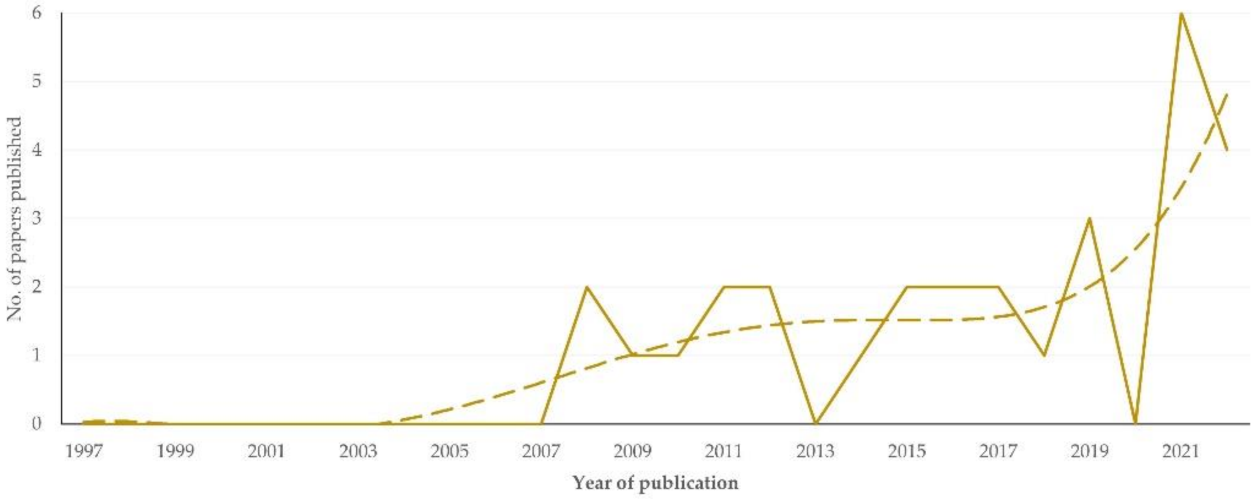

Table S5. Details of papers on sheep and goats in papers published during 1997 to 2022 in Greece, according to the topography and the nature of diseases described in the papers (in alphabetical order).

**Topography of disease(s) described in the papers**	**No. of papers**
Abdominal cavity	6
Cardiovascular system & blood	7
Ear, nose and throat	8
Endocrine glands	1
Eyes	1
Gastrointestinal tract	72
Genital system	41
Multi-system	93
Musculosceletal system	14
Nervous system	33
Respiratory system	14
Skin	28
Udder	79
Urinary system	6
**Nature of disease(s) described in the papers**	**No. of papers**
Bacterial	164
Ecto-parasitic	81
Endo-parasitic	23
Fungal	1
Genetic	1
Metabolic	20
Multi-faceted	27
Obstetrical	6
Prion-related	21
Surgical	7
Toxicosis-related	9
Viral	43

Table S6. Countries with establishments with which there was international collaboration in papers on sheep or goats published during 1997 to 2022 in Greece, according to the continent and the country of origin (in alphabetical order).

Country	No. of papers
Africa	
Egypt	7
Cameroon	2
Ethiopia	2
Algeria	1
America	
Unites States of America	19
Canada	3
Asia	
Jordan	14
Pakistan	4
United Arab Emirates	3
Iran	2
Turkey	1
Israel	1
Saudi Arabia	1
China	1
Europe	
United Kingdom	89
France	38
Italy	36
Cyprus	20
Germany	20
Switzerland	14
Spain	14
Belgium	12
Denmark	7
Austria	6
The Netherlands	6
Hungary	5
Serbia	3
Portugal	3
Norway	2
Czech Republic	2
Sweden	2
Ireland	2
Bulgaria	1
Slovenia	1
Poland	1

Russia	1
Oceania	
Australia	2
New Zealand	2

Table S7. Papers on sheep and goats published during 1997 to 2022 in Greece, in which there was international collaboration, according to the general topic of the study.

General topic of study	Number of papers	Percentage among all papers in the general topic
Health and welfare	170	33.7
Animal products	41	20.4
Production systems and sustainability	35	31.0
Genetics and breeding	26	26.0
Nutrition	24	23.8
Physiology	21	16.8
Human-related models	7	33.3
Society and professionals	4	25.0

Table S8. Measures of impact of papers on sheep and goats published during 1997 – 2022 in Greece, with origin from the four establishments with most papers.

Establishment	Total citations	Average citations per paper	*h*-index	i_{10}-index	Average yearly citations per paper
Aristotle University of Thessaloniki	6205	16.9	41	181	1.6
University of Thessaly	4403	14.5	31	145	1.6
Agricultural University of Athens	3438	15.0	29	106	1.8
Hellenic Agricultural Organization – Dimitra	2273	15.5	27	76	1.8
p value		0.49	0.08	0.78	0.54

Table S9. Proportion of papers on sheep and goats published during 1997 to 2022 in Greece with open access, according to the general topic of the study.

General topic of study	Percentage among all papers in the general topic
Health and welfare	30.1
Animal products	26.4
Production systems and sustainability	30.1
Genetics and breeding	35.0
Nutrition	22.8
Physiology	10.4
Human-related models	9.5
Society and professionals	37.5
